# Evidence for cortisol production in a shark

**DOI:** 10.1098/rspb.2026.1440

**Published:** 2026-10-14

**Authors:** Alyssa M. Weinrauch, Ian A. Bouyoucos, W. Gary Anderson

**Affiliations:** ^1^ Biological Sciences, University of Manitoba, Winnipeg, Manitoba, R3M 2N2, Canada; ^2^ Bamfield Marine Sciences Centre, Bamfield, British Columbia, V0R 1B0, Canada

**Keywords:** corticosteroids, elasmobranch, glucocorticoid, mineralocorticoid, *Rhodococcus*, high-performance liquid chromatography

## Abstract

Corticosteroids are essential regulators of numerous physiological functions in vertebrates, including energy balance, immune responses, osmotic balance and metabolism. The terminal corticosteroids are highly conserved across jawed vertebrates, where cortisol or corticosterone mediates these physiological responses. Elasmobranchs, however, have a unique dominant circulating steroid, 1⍺-hydroxycorticosterone, and since its discovery it has been presumed that elasmobranchs do not produce cortisol. Here, we demonstrate that cortisol is produced by North Pacific spiny dogfish (*Squalus suckleyi*). For the first time to our knowledge, we confirm the presence of transcripts encoding Cyp17 (which produces steroid intermediates of cortisol) in the interrenal tissue. We provide evidence of circulating cortisol in dogfish following two experimental methods: recovery from a hypoosmotic challenge (48 h at 21 ppt) and a 5-day gavage of antibiotics. Finally, cortisol implants at physiologically relevant levels were used to determine putative glucocorticoid and/or mineralocorticoid roles. Broadly, a persistent depression of plasma glucose occurred with implantation while little effect was seen on plasma osmolytes, yet definitive roles remain elusive. Together, we demonstrate context-dependent cortisol production in an elasmobranch and that endogenous cortisol at this level exerts a glucoregulatory effect. Further, our findings support a novel hypothesis for microbial steroid biosynthesis in elasmobranchs.

## 1. Introduction

Corticosteroid hormones are fundamental regulators of energy and mineral balance in jawed vertebrates [1]. Cortisol (F) and corticosterone (B) are the predominant circulating corticosteroids in all tetrapods [2] and teleosts [3], where they influence myriad physiological systems, including metabolism, immunoregulation, osmoregulation and energy balance. Notably, F and B are secreted under the control of the hypothalamus–pituitary–adrenal/interrenal (HPA/I) axis, which is a highly conserved feature of jawed vertebrates ( figure 1*a*; [4,5]).

**Figure 1.**
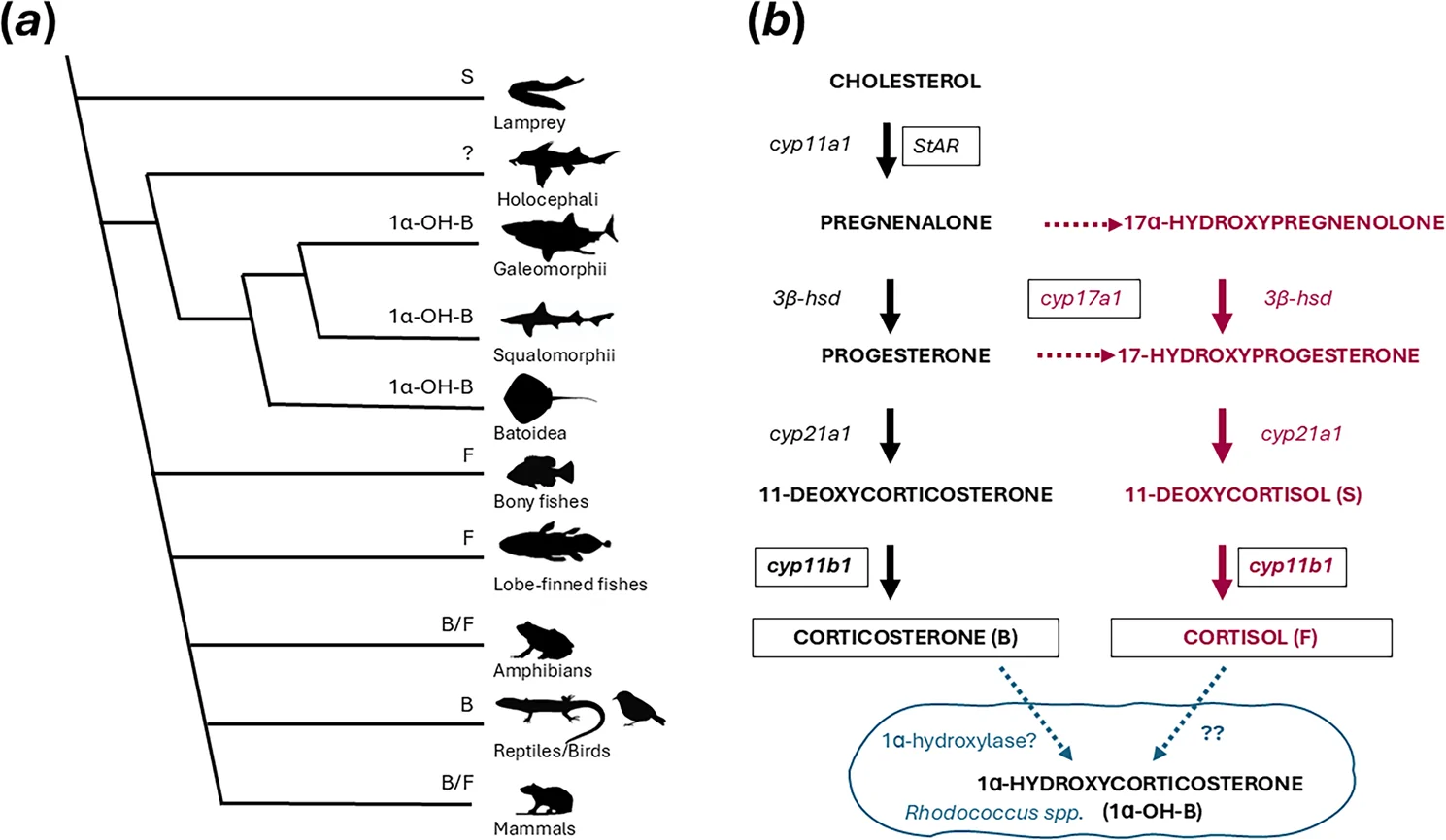
Corticosteroid biosynthesis pathways in vertebrates. (*a*) Vertebrate phylogeny, with emphasis on the elasmobranch superorders and known terminal corticosteroids affecting mineralocorticoid and/or glucocorticoid action. (*b*) The biosynthetic pathway of the unique elasmobranch corticosteroid, 1⍺-hydroxycorticosterone (1⍺-OH-B), is unknown and thought to occur via several mechanisms. Solid arrows denote confirmed pathways and dashed arrows are hypothesized. Steps shown in red represent the cortisol (F) synthesis pathway of teleosts and tetrapods, which remain unconfirmed in elasmobranchs. Blue cartoons represent putative bacterial contributions to 1⍺-OH-B synthesis. Steroids and transcripts in boxes were measured in this study. S, 11-deoxycortisol; B, corticosterone; StAR, steroidogenic acute regulatory protein.

Elasmobranch fishes (i.e. sharks and rays) are the earliest diverging jawed vertebrates and share components of a conserved corticosteroid synthesis pathway with teleosts and tetrapods [6]. Until the 1960s, circulating levels of F and B were reported in elasmobranchs (summarized in [7]). In 1966, Idler & Truscott described a novel corticosteroid, 1⍺-hydroxycorticosterone (1⍺-OH-B), in two species of ray (*Leucoraja ocellata* and *Amblyraja radiata*), and they further confirmed its presence in 19 elasmobranch species (representing Batoidea, Squalomorphii and Galeomorphii; [8–10]). Prior accounts of F in elasmobranchs were attributed to immuno-cross-reactivity with this unique steroid and it has since been presumed that F is not produced in elasmobranchs [8–10]. Attempts to resolve the biosynthetic pathway of 1⍺-OH-B corroborate a lack of F production via an inability of interrenal homogenates of seven species of shark to produce 17⍺-hydroxylated steroids *in vitro*, which suggests the absence of the cytochrome P450c17 enzyme (Cyp17; figure 1*b*; [11,12]). Because B and 1⍺-OH-B are nearly structurally identical, the most parsimonious synthesis pathway is the hydroxylation of C1 by a hypothesized 1⍺-hydroxylase [9].

Activity of 1⍺-hydroxylase is described across kingdoms, including within the vertebrate kidney [13] and in bacteria [14,15]. Early attempts did not find evidence of 1⍺-hydroxylase in the interrenal tissue of *Raja brachyura* [16], despite prior indirect evidence that 1⍺-OH-B is produced from B by interrenal homogenates [10]. More recent attempts to resolve the pathway described ureolytic bacteria of the genus *Rhodococcus* in the interrenal gland of winter skate (*L. ocellata*) and documented steroid catabolism of putative precursors of 1⍺-OH-B [17]. Bacterial genomic evidence did not identify a 1⍺-hydroxylase enzyme [18], and catabolism of known steroid precursors did not produce 1⍺-OH-B; however, catabolism of F produced an unknown steroid of nearly identical molecular weight and polarity to 1⍺-OH-B [17]. Again, an apparent lack of Cyp17 in the interrenal gland [11,12] precludes F as a relevant precursor of 1⍺-OH-B; however, molecular techniques have yet to be applied to identify this enzyme.

The biosynthetic pathway of 1⍺-OH-B is therefore hypothesized to occur by two mechanisms: hydroxylation of B by 1⍺-hydroxylase and/or steroidal catabolism by interrenal *Rhodococcus* spp. The latter hypothesis is, at present, complicated by an unresolved F synthesis pathway in elasmobranchs. Here, we propose that F synthesis occurs at low basal levels which could be masked by bacterial catabolism in the interrenal gland. To test this, we utilized molecular tools to screen for interrenal steroidogenic enzymes in the Pacific spiny dogfish (*Squalus suckleyi*). Then, we measured plasma steroids (following high-performance liquid chromatography (HPLC) separation) under two scenarios that are known to reduce bacterial communities in this species: (i) acute exposure to low salinity [19] and (ii) antibiotic cocktail gavage [20]. The intent with these protocols was to activate the HPI axis to affect steroid biosynthesis rather than induce a stress response (i.e. homeostatic overload *sensu* the reactive scope model [21]). Finally, we utilized F implants to elucidate a physiological role for F in dogfish.

## 2. Material and methods

### (a) Animal capture and housing

All experimental protocols were approved by the Bamfield Marine Sciences Centre (BMSC) animal care committee (RS-21-03) in accordance with the guidelines of the Canadian Council for Animal Care. Pacific spiny dogfish (*Squalus suckleyi*; 1.96 ± 0.45 kg; mean ± s.d.) were collected in July and August 2021 by hook-and-line or demersal longline off Brady's Beach, Bamfield, BC, Canada (48.827° N, 125.153° W) with a collection permit from the Department of Fisheries and Oceans Canada (no. XR-139 2021). Upon capture, animals were transported to the BMSC, where they were placed into an approximately 150 000 l tank of continuously flowing seawater (32 ppt, 11°C). Dogfish were fed Pacific hake (*Merluccius productus*) ad libitum every other day and fasted for at least 2 days before experiments.

### (b) Experimental protocols

#### (i) Dogfish interrenal transcriptome

Transcriptomic assessments were conducted on a previously assembled and annotated *S. suckleyi* interrenal reference transcriptome [22]. BLASTp/x hits were screened to identify *star*, *cyp11a1*, *3β-hsd*, *cyp17a1*, *cyp21a1* and *cyp11b1* transcripts. Further, we screened for evidence of bacterial transcripts belonging to the genus *Rhodococcus*.

#### (ii) Experiment 1: induction of cortisol production via acute exposure to low salinity

To induce F production, we first utilized a low-salinity challenge because this method has previously reduced ureolytic bacteria in *S. suckleyi* [19] and is an osmotic challenge that may invoke a mineralocorticoid response. Dogfish were captured from the large holding tank and a caudal blood sample was immediately drawn using a heparinized (50 IU ml^–1^) 18-gauge needle. Animals were indiscriminately assigned to approximately 5000 l tanks containing either full-strength (32 ppt) or diluted seawater (21 ppt) for 48 h, followed by transfer to recovery tanks (approx. 5000 l, 32 ppt) for up to 72 h. A subset of animals (*n* = 6 control, *n* = 7 low salinity) were euthanized in seawater containing 0.2 g l^–1^ tricaine methanesulfonate (Syndel Labs, Nanaimo, Canada) at 6 h of recovery and the interrenal gland was excised and immediately frozen under liquid nitrogen and stored at –20°C until further processing. The remaining animals (*n* = 5 low salinity) were serially blood-sampled at 2, 6, 12, 24, 36 and 72 h of recovery. All collected blood samples were immediately centrifuged (2 min at 13 000*g*) and the plasma was collected and frozen in liquid nitrogen before transport and further processing at the University of Manitoba.

#### (iii) Experiment 2: induction of cortisol production via repeated antibiotic cocktail gavage

Antibiotic cocktail administration was used as a secondary method to elicit F production as previous experiments have demonstrated reductions in ureolytic bacteria [20]. Dogfish in this experiment are the same individuals as in [20]. Briefly, dogfish were mildly anaesthetized and given daily gavage of either an antibiotic cocktail (in mg kg^–1^: 75 chloramphenicol, 75 ampicillin, 75 oxytetracycline, 20 sisomicin, 50 penicillin G; purchased from Drugs.com; *n* = 5) or seawater (volume-matched sham; *n* = 5), after which they were housed in approximately 5000 l tanks containing continuously flowing 32 ppt seawater. On the fifth day, a 2% ration of blended Pacific hake was included in addition to the antibiotics and the animals were placed into individual 40 l recovery boxes with constantly flowing seawater [20]. Caudal blood samples were drawn as above at 0 and 72 h of recovery and aliquots of plasma were frozen and stored in liquid nitrogen until further processing. After 72 h, animals were euthanized and the interrenal gland collected and stored as described above.

#### (iv) Experiment 3: physiological role of cortisol *in vivo*

To test for a physiological role *in vivo,* we utilized F implants and investigated endpoints related to glucocorticoid and mineralocorticoid actions. Dogfish were mildly anaesthetized and an initial (time = 0) blood sample was drawn as described above prior to an intraperitoneal injection of either 20 mg kg^–1^ cortisol dissolved in coconut oil or an equal volume of coconut oil alone (sham). Each group was revived in 32 ppt seawater and subsequently housed in approximately 5000 l tanks receiving continuously flowing seawater (*n* = 6 per tank). Caudal blood samples were drawn as above at the following timepoints post-injection: 0, 2, 6, 12, 24, 48, 72 h. Blood was processed and stored as described above.

### (c) Analysis

#### (i) Corticosteroids

Samples were extracted and corticosteroids separated based upon size, charge and hydrophobicity using HPLC separation as described previously [17]. Briefly, 750 µl of plasma was combined with 1.5 volumes of ice-cold acetonitrile (ACN) and vortexed for 30 s before centrifugation (12 000*g*) for 20 min at 4°C. The supernatant was collected, and the above process was repeated on the remaining pellet, after which the supernatants were combined and dried down under a gentle stream of nitrogen at 35°C, prior to storage at –20°C. Samples were reconstituted in 1 : 1 ACN : methanol (MeOH) and separated by HPLC on an Apollo C18 analytical column (Agilent Technologies) using a Gilson-Mandel Scientific HPLC system (506C system interface, 155 UV/VIS detector, 321 HPLC pump and GX-271 analytical liquid handler). Mobile phase (starting at 40% 1 : 1 ACN : MeOH and 60% water) was run at 1 ml min^–1^ for 10 min before 100 µl of sample was injected. A gradient of mobile phase was run for the next approximately 50 min with a final eluent concentration of 100% 1 : 1 ACN : MeOH. Retention times for B and F were confirmed using known concentrations of each steroid dissolved in mobile phase with UV detection at 254 nm. Further, a panel of steroids was run to confirm collection of individual steroids of interest within a specific fraction. Fractions were collected every 1.5 min using an autosampler, and immunoreactivity of B and F was tested (see below) in each fraction to confirm the presence of steroid (supplementary material, figure S1a,b). Standards were periodically injected among samples to ensure there was no drift in retention time of targeted steroids. Upon collection, fractions were dried down under a gentle nitrogen stream at 35°C, during which time fractions 7–9 and 11–13 were combined as they displayed immunoreactivity with F and B, respectively. Dried samples were stored at –20°C until measurement. Notably, fractions 2 and 3 had an evident chromatogram peak and showed low immunoreactivity with B, suggesting that these fractions could contain 1⍺-OH-B as previous studies have found a similar retention time using this protocol [23]. However, owing to a lack of available 1⍺-OH-B standard, we were unable to confirm this.

B was measured in duplicate in pooled fractions using a commercial enzyme immunoassay (EIA; Cayman Chemical Company), following previously described methods [23]. Samples that exceeded a coefficient of variation of 20% were rejected and re-assayed. Serial dilution of samples displayed parallelism, and extraction efficiency was calculated as 90.4 ± 10.1%. F was measured in duplicate by means of a previously described radioimmunoassay (RIA) [24] using a 1 : 15 000 cortisol-specific antibody dilution (Perkin-Elmer, Waltham, MA, USA) with a binding efficiency of approximately 30%. When F concentration was below that of %B/B_0_ of the lowest standard (i.e. 0.2 ng ml^−1^), the sample was re-extracted and re-run. If still below this limit, the value was considered below detectable limits and was not included in the analysis. Extraction efficiency was variable at 87.2 ± 25.9%. Owing to the low concentration of F within the samples, parallelism could not be determined as any dilution of samples, even those combined and concentrated, led to undetectable levels of F. Finally, the presence of F within two independent samples was verified with liquid chromatography paired with tandem mass spectrometry (LC-MS/MS; supplementary material, S1, figure S2*a*–*c*).

**Figure 2.**
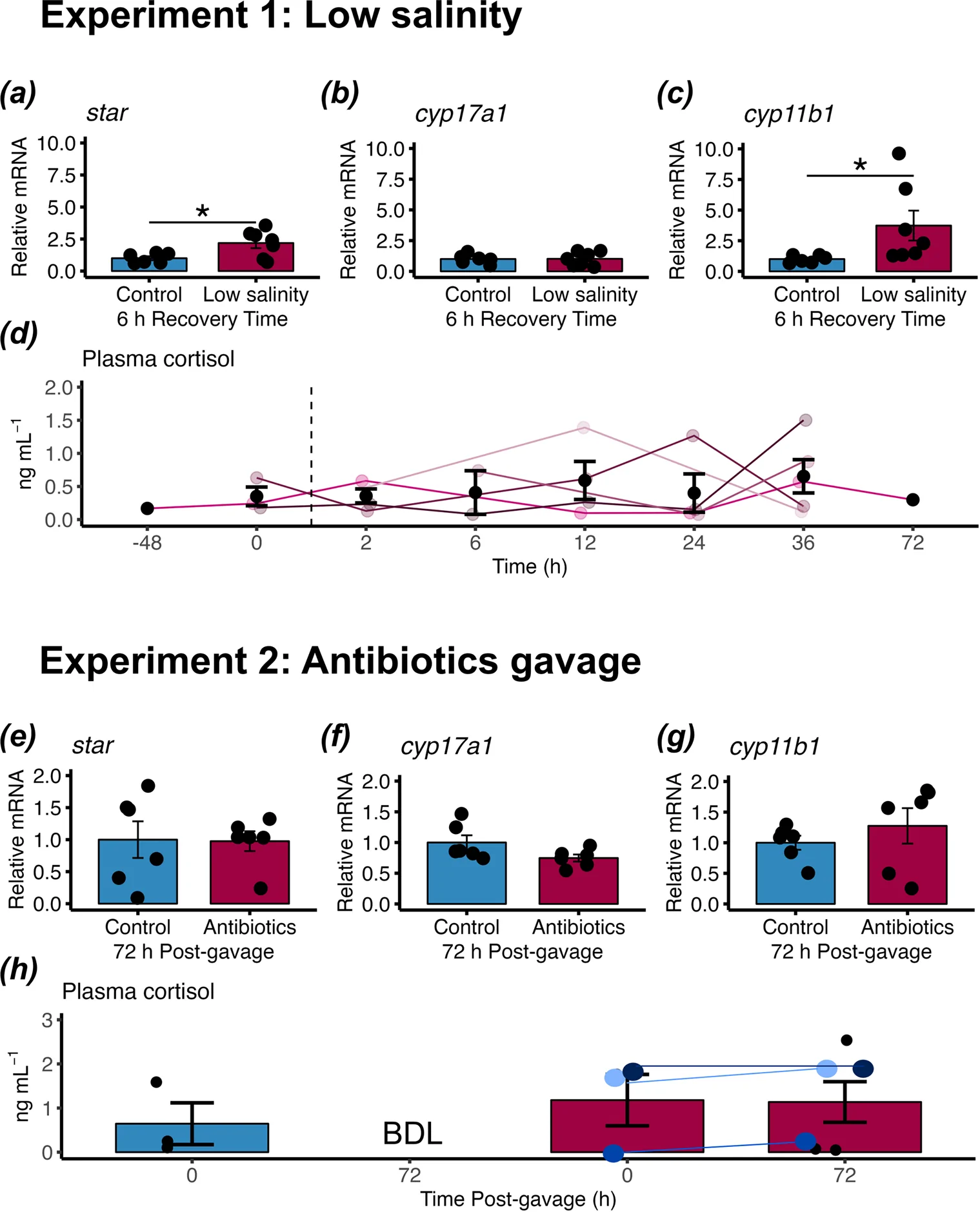
Evidence of cortisol synthesis in Pacific spiny dogfish (*Squalus suckleyi*) under two experimental conditions. In experiment 1, dogfish were exposed to 32 (blue) or 21 (red) ppt seawater for 48 h (left of the dashed vertical line in (*d*)) and then recovered in 32 ppt for 6 or 72 h. Interrenal tissue was sampled after 6 h of recovery to measure relative mRNA abundance of key components of the corticosteroidogenic pathway (see figure 1), including steroidogenic acute regulatory protein (*star*; *a*), *cyp17a1* (*b*), and *cyp11b1* (*c*). Plasma was serially sampled and separated by high pressure liquid chromatography (HPLC) to measure cortisol by radioimmunoassay (*d*). In experiment 2, dogfish were anaesthetized and received daily gavages of seawater (blue) or an antibiotic cocktail (red) for 96 h followed by 72 h recovery. Interrenal tissue was sampled after 72 h of recovery for *star* (*e*), *cyp17a1* (*f*), and *cyp11b1* (*g*). Plasma was collected immediately after gavage (0 h) and following 72 h recovery (*h*) and HPLC-separated prior to F measurement by radioimmunoassay. Data are presented as means ± s.e.m. with individual data points shown for *n* = 5–7 animals. Asterisks denote significant differences as determined by unpaired *t*-tests with *p* < 0.05. No detectable plasma cortisol was observed at timepoint 72 in sham-gavaged dogfish (*h*). Repeated measures of individual dogfish are indicated by colour-coded dots and lines (d and h). BDL, below detectable limit.

#### (ii) Plasma osmolytes and metabolites

Plasma osmolality was measured by vapour pressure osmometry (Wescor 5100C; Wescor, Logan, UT, USA), while plasma urea was measured using the diacetyl monoxime method [25] on diluted samples. Plasma was appropriately diluted and glucose was measured using the hexokinase method [26], while β-hydroxybutyrate (β-HB) was measured using a β-hydroxybutyrate dehydrogenase method [27]. All assays were run in duplicate in clear flat-bottomed 96-well plates on a BioTek Powerwave spectrophotometer (VT, USA).

#### (iii) Interrenal molecular analyses

Total RNA was extracted from interrenal tissue using TRIzol (Ambion Life Technologies) according to the manufacturer's specifications. Integrity and quantity were verified using gel electrophoresis and a NanoDrop spectrophotometer, respectively. Following DNAse1 treatment (Invitrogen, CA, USA), cDNA synthesis was performed using iScript (Bio-Rad Laboratories, CA, USA) as specified by the manufacturer and stored at –20°C. Gene-specific primers (*star*, forward (for.): GAATGGACCCACTTGCATTGT, reverse (rev.): GATCAGCAGCCATGTGAACTTG; *cyp17a1*, for.: TGGGCTATTCACCACGATGAA, rev.: GTGTTTCCCATCCTCATCCAA; *cyp11b1*, for.: GTACCCCATCGGCGTCACT, rev.: CCGGCTGGAATGTGGTAATT) were designed from the *S. suckleyi* interrenal transcriptome using PrimerExpress (v. 3.0.1). Reference genes were obtained from published sources (*actb*, for.: TGCACTGGACTTTGAACAGG, rev.: TTCCACAGGATTCCATACCC [28]; *rpl7*, for.: CGCAAACTATACAGTGGGACCT, rev.: AAATTGGGAAACCCCCAGGC [29]). Quantitative real-time PCR (qPCR) was performed in 96-well plates using a BioRad CFX Connect qPCR Machine. Primer efficiency validations were determined by serial slope dilution calculation, where slopes of 0 ± 0.1 were determined equally efficient and suitable for analysis (supplementary material, figure S3). Individual reaction mixtures consisted of 5 µl SYBR Green master mix (Bio-Rad Laboratories), 2.5 µl diluted complementary DNA (cDNA) and 2.5 (*actb, star*) or 1.25 µl (*cyp11b*,* cyp17a1*,* rpl7*) of primer mix (3.2 µM), with nuclease-free water added where needed to a total of 10 µl. Cycling conditions included 10 min of initial denaturation at 95°C followed by 30 cycles of 95°C for 30 s and 60°C for 15 s, with a melt curve analysis added after the final cycle. Transcript changes were determined by ΔΔ*C*_T_ analysis using *actb* or *rpl7* as an endogenous control [30]. The stability of endogenous controls was assessed in all treatment groups, with an average difference of <0.5 *C*_T_ value required to be considered stable.

#### (iv) Data visualization and analysis

Data were analysed and visualized using RStudio (v. 2023.09.1+494; [31]) with significance set as *p* < 0.05 for all analyses. Normality of paired analyses (e.g. qPCR results, all antibiotic measurements) was assessed using the Shapiro–Wilk normality test. All datasets were normal and assessed with an unpaired *t*-test, except for one (low-salinity *cyp11b1*), which was assessed with a Wilcoxon rank sum exact test. A linear mixed-effects model fitted with the ‘*nlme*’ package [32] was used for comparisons of F and B by time, with individual animals assigned as a random factor. This method was also used for plasma osmolality, urea and glucose in low-salinity-exposed fish. F values from the low-salinity treatment were log-transformed to conform to assumptions of normality and homogeneity of variance as determined by Shapiro–Wilk and Levene's tests, respectively. The single values at the start (–48 h) and end (72 h) of the time course were not included in the statistical analysis. In the antibiotic gavage experiment, plasma F values were assessed using a two-way analysis of variance (ANOVA) with treatment and time as nominal factors; however, owing to zero values, the 72 h time point in the sham group was not included in this model. We also compared treatment effects regardless of time using a Wilcoxon test. Plasma constituents from implanted animals were analysed using a linear mixed-effects model where the measured parameter was modelled as a function of the interaction of ‘treatment’ and ‘time,’ with animal ID assigned as a random factor. Linear model assumptions were checked as above. *Post hoc* multiple comparisons were made by calculating least-squares means within the ‘*lsmeans*’ package [33]. Raw data are publicly accessible [34].

### (d) Results

#### (i) Dogfish interrenal transcriptome

Transcripts of all genes required for corticosteroid synthesis, including *cyp17a1*, were identified within the *S. suckleyi* interrenal reference transcriptome. *cyp17a1* had 98.9% identity with the previously identified gonadal *cyp17a1* of *S. acanthias* (supplementary material, figure S4; [35]). Numerous genes related to translation initiation factors attributed to the genus *Rhodococcus* (12 transcripts representing four genes) were identified within this tissue.

#### (ii) Induction of cortisol production via acute exposure to low salinity

Predicted effects on plasma osmolality, urea and glucose occurred during and following acute low-salinity exposure (supplementary material, figure S5a–c). After 6 h of recovery from acute exposure to low salinity, significant increases in *star* (*t* = –2.78, d.f. = 7.7, *p* = 0.025) and *cyp11b1* (*W* = 2, *p* = 0.005) mRNA abundance were observed, when compared with controls; however, no transcriptional response was noted for *cyp17a1* (*t* = –0.06, d.f. = 10.53, *p* = 0.96; figure 2*a*–*c*). Detectable F was found in the plasma of only one dogfish prior to low-salinity exposure (–48 h) and at 72 h of recovery in full-strength seawater, while variable levels of detectable F were measured from the end of the acute exposure up until 36 h of recovery. Over this time course, no significant differences in average F (*F*_11_ = 0.37, *p* = 0.86; figure 2*d*) or B (supplementary material, figure S6a; *F*_17_ = 1.51, *p* = 0.23) concentrations were noted.

#### (iii) Induction of cortisol production via repeated antibiotic cocktail gavage

No differences in mRNA abundance of the selected steroidogenic genes *star* (*t*(7.71) = 0.07, *p* = 0.94), *cyp17a1* (*t*(7.28) = 1.93, *p* = 0.09) or *cyp11b1* (*t*(6.5) = –0.89, *p* = 0.41) were found between saline-gavaged dogfish and those gavaged with an antibiotic cocktail (figure 2*e*–*g*). Notably, only three of six (50%) control individuals had detectable F, while the hormone was detected in six of six (100%) antibiotics-gavaged individuals; however, no treatment-specific effects were noted for F (as assessed by both Wilcoxon test on summary of treatments (*W* = 10, *p* = 0.6) or ANOVA (*F*_1_ = 0.53, *p* = 0.48); figure 2*h*) or B (supplementary material, figure S6b; time: *F*_1_ = 0.85, *p* = 0.37, treatment: *F*_1_ = 3.61, *p* = 0.08, interaction: *F*_1_ = 0.001, *p* = 0.97).

#### (iv) Physiological role of cortisol *in vivo*

As expected, plasma F was significantly increased with F-implantation compared with sham controls (*F*_1,43_ = 53.97, *p* < 0.0001; figure 3*a*), with concentrations decreasing over time (*F*_5,43_ = 2.51, *p* = 0.04), but no interactive effect of time and treatment (*F*_5,43_ = 0.85, *p* = 0.52). Detectable F within the sham-treated individuals is attributed to induction of a stress response following surgery and implantation. This argument is further strengthened by undetectable F levels within all dogfish at the pre-injection baseline sampling point (time = 0). The B response that was evident in sham-implanted dogfish was abolished in F-implanted fish (treatment: *F*_6,56_ = 0.29, *p* = 0.59; time: *F*_1,56_ = 6.22, *p* < 0.0001; interaction: *F*_6,56_ = 2.32, *p* = 0.046; figure 3*b*). Plasma glucose was not significantly altered over the 72 h time course, but a noted global suppression occurred in F-implanted fish (treatment: *F*_1,63_ = 10.19, *p* = 0.002; time: *F*_6,63_ = 1.38, *p* = 0.24; interaction: *F*_6,63_ = 0.95, *p* = 0.47; figure 3*c*). There were no significant effects of treatment on β-HB (treatment: *F*_1,10_ = 0.07, *p* = 0.80; time: *F*_1,51_ = 1.79, *p* = 0.12; interaction: *F*_6,51_ = 0.94, *p* = 0.48; figure 3*d*), osmolality (treatment: *F*_1,10_ = 0, *p* = 0.97; figure 3*e*) or urea (treatment: *F*_1,61_ = 3.07, *p* = 0.08; figure 3*f*). A significant interaction was noted for plasma urea, where an elevation was noted at 24 h in F-implanted fish (*F*_6,61_ = 2.32, *p* = 0.04). A significant and transient increase in plasma osmolality was also noted at 2 h (*F*_6,58_ = 34.66, *p* < 0.0001), but no interactive effect was observed (*F*_6,58_ = 1.44, *p* = 0.22).

**Figure 3.**
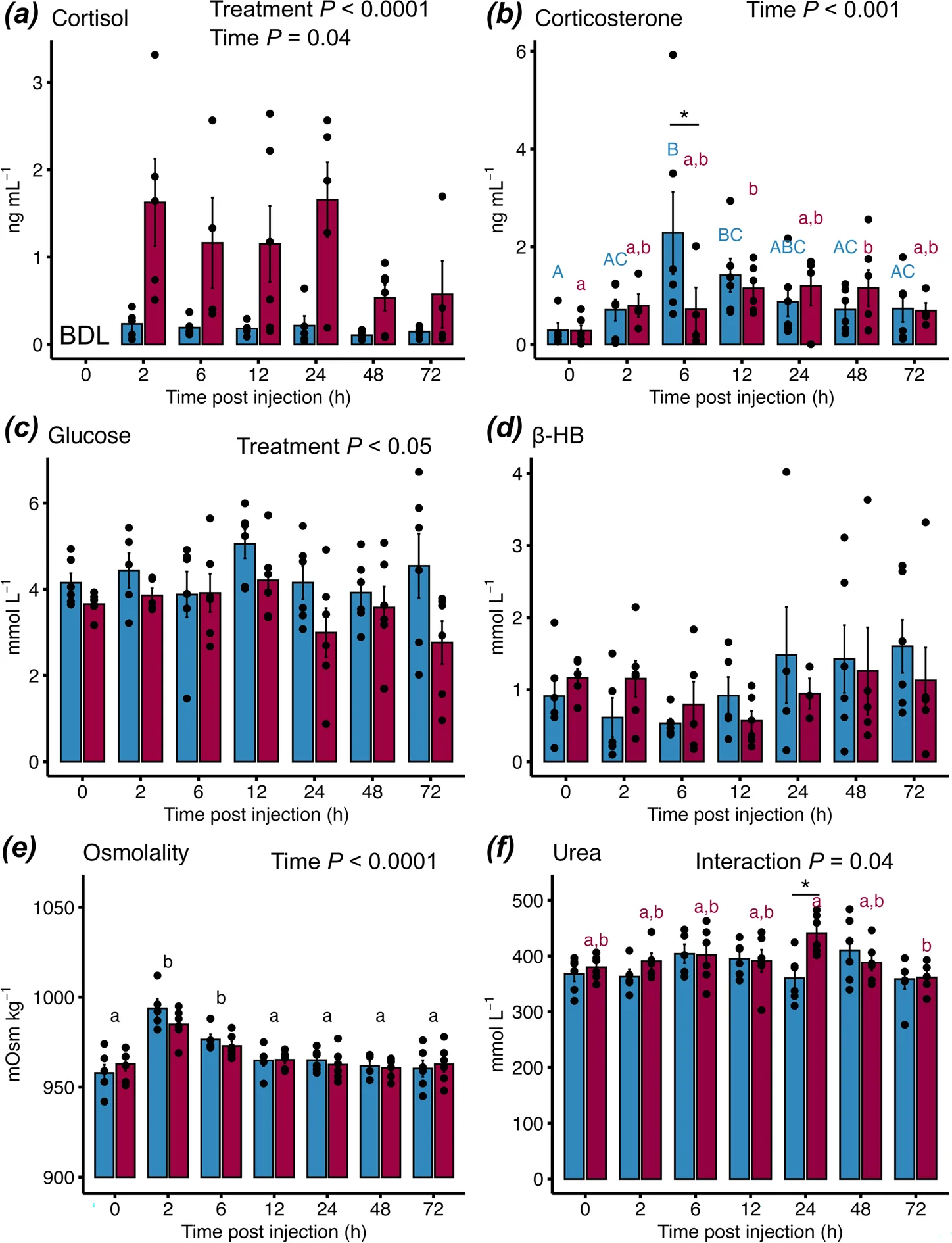
Effects of cortisol implantation on plasma parameters of the Pacific spiny dogfish (*Squalus suckleyi*) over 72 h. Following implantation with sham (blue) or 20 mg kg^–1^ cortisol (red), dogfish plasma was measured for cortisol (*a*), corticosterone (*b*), glucose (*c*), β-hydroxybutyrate (β-HB; *d*), osmolality (*e*) or urea (*f*). Note: data collected at time 0 are pre-injection baseline controls. Data are presented as means ± s.e. with individuals shown. Different letters denote time-dependent differences, while the asterisk denotes a significant treatment effect at that time (*p* < 0.05). Significant effects of treatment, time or interaction are noted on the individual graph, with a corresponding *p*-value. BDL, below detectable limits.

## 3. Discussion

Evidence since the 1960s–1970s has supported the prevailing notion that elasmobranchs cannot produce F [8–10]. Through a combination of classical chromatography and modern molecular biology techniques, the present study demonstrates biosynthesis of F in the Pacific spiny dogfish, presumably by a conserved steroidogenic pathway via a 17⍺-hydroxylase. Further, we provide evidence of an ecological context-dependence for F synthesis *in vivo*.

Our study provides the first direct molecular evidence for the presence of *cyp17a1* within the interrenal tissue of an elasmobranch, supporting a conserved F synthesis pathway. This finding supports a mechanism by which F synthesis in *S. suckleyi* can occur, via 17⍺-hydroxylation of progesterone and pregnenolone; a confirmed function of Cyp17 previously isolated from *S. acanthias* gonadal tissue [35]. Previous studies suggested that elasmobranchs lack interrenal Cyp17a1 because homogenates of this tissue did not synthesize 17⍺-hydroxylated steroids, including interrenal homogenates from the congener of *S. suckleyi*, *S. acanthias* [11,12]. From a phylogenetic perspective, it would be curious for elasmobranchs not to express Cyp17a1 in interrenal tissue because the cyclostomes (which diverged from the vertebrate lineage prior to elasmobranchs) are predicted to express a Cyp17 gene in their steroidogenic tissues to synthesize 11-deoxycortisol [1,36]. Regardless, now given its molecular confirmation in this tissue, there is a need for studies to characterize the activity of *S. suckleyi* Cyp17a1. We did not observe elevations in *cyp17a1* mRNA abundance under conditions where *S. suckleyi* were producing more F relative to control treatments; however, this finding may suggest that endogenous Cyp17a1 is present at an abundance that is capable of modulating steroid production (notably, not just F but other steroids; see figure 1*b*) prior to induction of the stress response. Alternatively, time-dependent changes in transcription may have been missed given our single sampling time point. Future studies could address the functional relevance of this transcript as it relates to osmotic challenges by conducting a sampling regime across multiple time points before, during and after the challenge. On the other hand, it has been previously shown that genes/enzymes involved in the regulation of the vertebrate stress response may not be transcriptionally responsive following acute stress *in vivo* or *in vitro* [37].

F synthesis follows a conserved steroidogenic pathway in vertebrates; thus, we examined the steroid biosynthetic pathway by investigating *star*, a gene encoding a transport protein that mediates cholesterol entry into this pathway [38], and * cyp11b1*, the gene for the terminal enzyme responsible for both F and B production [39]. Following 6 h recovery from acute low-salinity exposure, significant increases in mRNA abundance of *star* and *cyp11b1* were noted, suggestive of an increase in steroid biosynthesis. While elevated *star* and *cyp11b1* transcript abundance coincided with increases in circulating F and B, repeated gavage did not. It is possible that the repeated daily anaesthesia and gavage in both sham- and antibiotics-treated groups led to induction of a stress response, thereby diminishing any potential differences in transcript and hormone endpoints. However, *star* and *cyp11b1* are both essential for the production of other steroids, including 1⍺-OH-B and B, and alterations to transcriptional abundance cannot be attributed to biosynthesis of any particular steroid, particularly as we were unable to measure 1⍺-OH-B here.

Biosynthesis of F was achieved *in vivo* following an acute osmotic challenge. Both experimental protocols revealed significant differences in the percentage of individuals with detectable F in their plasma. Prior to low-salinity exposure (i.e. true control animals), plasma F was below the lowest concentration in our standard curve (0.2 ng ml^–1^) in all but one individual, which is consistent with all prior reports which did not detect F in 19 elasmobranch species [8–10], leading to the conclusion that these fishes cannot synthesize F. After spending 48 h at reduced salinity, F was measured in 40% of individuals, and by 36 h of recovery F was detected in 100% of dogfish. By 72 h of recovery, F was once again undetectable in all but one dogfish, which suggests recovery from an acute osmotic challenge in this marginally euryhaline species and could indicate a decrease in F production or an increase/restoration in microbial F modification.

Treatment with antibiotics increased the detectability of circulating F in *S. suckleyi*. Contrary to our predictions, 50% of individuals had measurable F after 5 days of repeated gavage with both seawater and antibiotics. Following 72 h of recovery, 0% of seawater-gavaged dogfish had detectable F in their plasma, whereas 100% of antibiotics-gavaged dogfish had circulating F. It is possible that after 72 h of recovery, F levels could return to below detectable limits in the sham-gavaged dogfish, whereas the gavage of antibiotics exacerbated the stressor, leading to prolonged detection of F throughout that 72 h. Alternatively, it could implicate microbial influence on plasma steroid concentrations (i.e. microbial metabolism of F under sham conditions is not evident following antibiotics gavage, as the bacterial population may be dysregulated). Indeed, the aim of these two experimental protocols was to release F into circulation by reducing bacterial communities that could be catabolizing F within interrenal tissue. Previously, we have demonstrated that low-salinity exposure and antibiotic gavage reduce microbial enzyme activity (i.e. urease and cellulase) within the gut tissue [19,20]; however, it is unclear whether microbial communities in other tissues are affected. Attempts were made to measure microbial enzyme activity within the interrenal tissues in this study, but the lipid-rich nature of this tissue confounded results. However, recent collaborations have identified bacteria at the genus level derived from interrenal tissue of *S. suckleyi* (J. MacPherson 2026, personal communication). Future studies will use standard labelling techniques such as fluorescent *in situ* hybridization to locate and quantify these bacteria. Thus, it remains uncertain whether detection of F in circulation is a result of an impaired microbial community or a corticosteroid response itself. Interestingly, the increased detectability of F in antibiotics-gavaged fish and in fish recovering from an acute osmotic challenge lends credence to the hypothesis that interrenal bacterial communities may be involved in regulating corticosteroid catabolism.

Notably, the reported F concentrations (<2 ng ml^–1^) are quite low yet are within the resting range of another early-diverging vertebrate class, the chondrosteans (i.e. sturgeons) [40,41]. The presence of a second dominant corticosteroid, 1⍺-OH-B, may also influence resting circulating levels of F. *In vitro* receptor binding activation experiments reveal that F is a higher-affinity ligand than 1⍺-OH-B of the little skate (*Leucoraja erinacea*) mineralocorticoid (1.04 versus 3.84 nM) and glucocorticoid (139 versus 947 nM) receptors [42], further diminishing the need for elevated levels of F in circulation. We next sought to identify functional roles for this hormone at this physiological level. F implantation (at 20 mg kg^–1^) successfully increased circulating F from undetected to values reflective of those evoked by low-salinity exposure and gavage (i.e. approx. 1–3 ng ml^–1^). An expected transient increase in plasma B indicative of a stress response was observed within sham-implanted individuals, while plasma B concentrations were significantly suppressed 6 h post-surgery in F-implanted dogfish. Similar suppression of plasma steroid concentration following administration of a synthetic steroid, dexamethasone, has been identified in *Scyliorhinus canicula* [43,44]. This may be an indication of negative feedback regulation wherein steroid binding of the glucocorticoid and/or mineralocorticoid receptor can control the HPA/I axis to fine-tune both steady-state and acute stressor responses [45]. The specific role of each of these receptors in regulating the stress response in elasmobranchs remains to be determined.

The plasma concentrations of F in implanted dogfish (i.e. approx. 1–3 nM) are around the calculated EC_50_ for F activation of the little skate (*L. erinacea*) mineralocorticoid receptor (1.04 nM) and well below that calculated for the glucocorticoid receptor (139 nM; [42]). If the EC_50_ range is comparable across elasmobranchs, this could suggest activation of the mineralocorticoid receptor in this experimental paradigm. Yet, based on the data presented, it is difficult to ascribe a mineralocorticoid or glucocorticoid role to F. There was no impact of F implantation on plasma osmolality (a general measure of osmotic status) and only a transient effect on plasma urea (an important osmolyte in marine elasmobranchs; [46]). Specifically, plasma osmolality transiently increased at 2 h in both sham- and F-implanted individuals, consistent with a surgically induced stressor. Such increases in osmolality may result from alterations to osmolytes and metabolites as a result of the secondary stress response or could be an effect of the anaesthesia, which has been shown to influence plasma sodium in fishes [47,48]. Plasma urea was elevated at 24 h after F implantation compared with the paired control. This corresponds with the final time point where F values are elevated (i.e. some individuals reading >1 ng ml^–1^) and may hint at prolonged F elevation leading to alterations in urea regulation. Previously, repeated dexamethasone injection (0.5 mg kg^–1^) did not alter osmolality or plasma urea in *S. canicula* after 4 days [44]. Thus, a relationship between F and plasma osmolytes like urea, if it exists, remains to be explicitly determined in elasmobranchs. Of interest, 1⍺-OH-B has noted mineralocorticoid effects. For instance, an inverse relationship between plasma urea and 1⍺-OH-B occurs following low-salinity challenge in *S. canicula* [49]. Further, an osmoregulatory hormone, angiotensin II, plays a role in regulating 1⍺-OH-B synthesis in multiple elasmobranch species [50,51]. Perhaps this highlights the ability of F to exert glucoregulatory actions, while a glucocorticoid role for 1⍺-OH-B remains equivocal.

An overall reduction in plasma glucose concentration was noted for F-implanted fish compared with sham controls. This is consistent with reduced plasma glucose throughout low-salinity exposure until 72 h, when F was no longer detected and when glucose rose significantly. This may suggest that F could reduce gluconeogenesis or glycogenolysis or is promoting glucose utilization, thereby maintaining plasma concentrations. This speculation is complicated by existing studies wherein daily injection of 10 mg kg^–1^ F for 10 days led to hyperglycaemia in *S. suckleyi* [52], while 7 days following dexamethasone implantation (40 mg kg^–1^), plasma glucose levels were not altered in *S. canicula* [43]. This extended dexamethasone treatment also led to increased glycogen deposition in the liver and reduced glycogen storage in the muscle, suggestive of glucose liberation [43]. Explant experiments of these two tissues, however, reveal similar patterns of increased glucose secretion in the short term (1 h), but decreased glucose secretion over time (up to 5 h) [43]. Clearly, the role of glucose and its potential steroidogenic regulation in elasmobranchs remains ambiguous and is likely complicated by the fact that glucose is not relied upon as an energetic fuel in some elasmobranch tissues [46]. Given the reliance upon ketone bodies in certain tissues and physiological scenarios, we examined the ketone body β-HB [46]. There was no observed effect of F on β-HB; however, prior research examining numerous hormones, including B, and their effects on energy balance and mobilization has highlighted tissue-specific β-HB regulation that does not always correspond to a glucoregulatory effect [53–55]. Future studies pursuing the effect of F on elasmobranch metabolism are necessary to parse out putative tissue- and context-specific actions of this hormone. Finally, if circulating F levels were sufficiently high to activate the mineralocorticoid receptor but not glucocorticoid receptor, it is possible that activation of the mineralocorticoid receptor promotes glucose uptake by the skeletal muscle which is realized as our observed decrease in plasma glucose. Indeed, such a role for the mineralocorticoid receptor has been demonstrated via corticosteroid receptor knockout zebrafish models [56,57].

Our measurement of circulating F in *S. suckleyi* generates questions regarding the phylogenetic diversity of F production across elasmobranchs and the functional evolution of steroid hydroxylases in jawed vertebrates. Phylogenetically, it is salient to determine whether interrenal Cyp17a1 is retained in Squalomorphii but lost in galeomorph sharks and batoids; indeed, this is of particular relevance among euryhaline and obligate freshwater elasmobranchs, where reduced salinity may result in different responses from those observed herein with a marginally euryhaline species. Further, the presence of a 17⍺-hydroxylase in the steroidogenic tissues of cyclostomes (e.g. lamprey) and teleost fishes [58] brings into question its absence in interrenal tissues in other elasmobranchs. Indeed, Cyp17a1 is notably absent in the interrenal and adrenal tissue of most tetrapods (i.e. some amphibians and mammals, all reptiles and birds) that predominantly secrete B, which is a steroid precursor to the tetrapod mineralocorticoid aldosterone [59].

Our data provide indirect support for a novel hypothesis of steroid biosynthesis in elasmobranch fishes wherein bacteria contribute to steroidogenesis [17]. Specifically, we find support for two predictions of this hypothesis. First, using molecular tools, we corroborated reports of *Rhodococcus* spp. in the interrenal tissue of elasmobranchs, confirming the presence of prokaryotes with the capacity to biotransform steroids. Second, we demonstrated that exposing dogfish to conditions (i.e. hypoosmotic challenge and antibiotics treatment) that predictably reduce the abundance of bacteria that can metabolize F was associated with elevations in circulating F. While investigation into the mechanisms of steroid biosynthesis in elasmobranchs and the commensal (or symbiotic) nature of bacterial communities within elasmobranch interrenal tissue warrant further interrogation, our data unequivocally demonstrate that the North Pacific spiny dogfish synthesizes F.

## Supplementary Material



## Data Availability

Raw data are publicly available at Mendeley Data [34]. Supplementary material is available online [60].
