# Supplementary material for: Evidence for cortisol production in a shark
Source: Proc Biol Sci. 2026 Oct 14;293(2080):20261440. doi: 10.1098/rspb.2026.1440 (PMC13678717; doi:10.1098/rspb.2026.1440)
Supplement: Supplementary file 1 [file rspb.2026.1440.zip › rspb.2026.1440/rspb20261440_si_001.docx]

**Supplemental Figures**

**SI Figure 1. Verification of HPLC separation and antibody specificity of B and F.** *(****a****)* Trace demonstrating the elution time of a dogfish plasma sample spiked with 50 µM F and 50 µM B. Arrows identify fractions where these corticosteroids eluted as verified by single separate runs with each individual standard. A large and consistent peak was observed in all plasma samples in fraction 2 that we predict to be 1α-OH-B. *(****b****)* Corticosterone concentration in each eluted fraction using the ELISA kit demonstrated cross-reactivity only in fractions 12 (corresponding to the fraction where B eluted) and 2 (predicted to be 1α-OH-B). *(****c****)* Cortisol measures in each eluted fraction using the RIA demonstrates the greatest reactivity in fractions 8-10.

**SI Figure 2.** Chromatograms of F identified using LC-MS/MS in (***a***) cortisol standard, (***b***) sample from a dogfish exposed to low salinity for 48 h and recovered 24 h, (***c***) or 36 h in full-strength seawater. Dogfish plasma samples were extracted as described and reconstituted in acetonitrile:methanol (50:50, *v/v*) and filtered to 0.2 µm using a PTFE filter. Samples (2 µL) or cortisol standard (1 µL) were injected at room temperature into the LC-ESI-MS (Bruker Compact QTOF; Bruker, Billerica, MA) equipped with a C-18 Synergi 4 µm Polar-RP (50 x 2.00 mm) column and an electrospray source operating in positive ionization mode. Mobile phase (Solvent A: 0.1% formic acid in water, Solvent B: 0.1% formic acid in acetonitrile:methanol [40:60]) was injected at a flow rate of 0.5 mL min^-1^ with 30% Solvent B for 0-3 min before ramping linearly to 95% Solvent B. After 6 min at 95% Solvent B, the gradient was linearly ramped down to 30% Solvent B where it was held for a further 4 min prior to re-equilibration for the subsequent sample. The MS full scan was monitored from a 50 to 700 mass to charge ratio (*m/z*) in the positive ion mode. Data processing was performed using Bruker Compass Data Analysis software (v.5.2).

**SI Figure 3. Primer efficiency validations for quantitative real-time PCR**. Target genes were normalized to endogenous control genes across cDNA dilutions.

> *Squalus suckleyi cyp17a1*

ATGTGCTCCACATTTGAACGCTTGAACAACGCAGCGCACGACATGATGCCCGATGTGACACGAGCAGTCACCAATGTAATCTGCTTGCTGTGCTTCAACTCAACCTATGAGAAAGAAGACCCAGAGTTCCAGACCATGCGCAAGTACAGCCAAGGCATTGTGAACACTGTGGCAAAGGATAGCTTAATAGACATCTTCCCCTGGCTCCAGTTTTTTCCCAATGAAAATCTCCATACTCTGAAACAATGCATCGCAACTCGAGACAGTATTCTTCAGAAGAAATTTGAAGACCACAAGGCAAATTACAGCAGTGACTCTGCAAATGATTTGTTCAACATTCTGCTAAAAGCCAAGATGAATGCAGAGAACAACAACAGTTCTGTGCATGAGGCGGGACTGACGGACGACCACATGGTTATGACAGTGGCAGACATATTTGGGGCAGGGGTGGAGACCTCGAGCACGGCGTTTGCGTGGATGATTATTTATCTGATTCACCATCCTGAGGTGCAGAAAAAAATACAAGAGGAAATTGATAGCAACATCGGATTTGAAAGGACTCCCAAAATGAGTGATAAAGGCAATATGAATTTTCTCAATGCAACTATCCATGAAATCCTACGGATCCAACCAGTATCACCTCTCTTAATTCCACACGTGGCTTTGGCAGACTCTAGCATTGGTGACTACACAATCCCCAAAGGAACTCGTGTAATCATCAACCTCTGGGCTATTCACCACGATGAAAAGGAATGGAAGAACCCCGATGCCTTTGATCCAGGCCGATTTTTGGATGAGGATGGGAAACACGTCTGCTCTTCTTCGGAAAGTTACCTGCCTTTTTGGGGCGGGCACCAGGGTTTGCCTCGGCGAGATGCTGGCGAGGATGGAACTTTTTCTCTTCACGTCCTGGATTCTTCAGCGCTTCACGGTCCAAGTTCCCCCGGGTTATCCCCCGCCGGA

**SI Figure 4. North Pacific spiny dogfish (*Squalus suckleyi*) *cyp17a1*.** The nucleotide sequence for *S. suckleyi* *cyp17a1* was identified from screening a reference transcriptome of the interrenal gland (see ref. 21). The nucleotide sequence is also found in the Puget Sound Dogfish (*S. suckleyi*) genome assembly (GSC_Ssuck_1.0) on NCBI under isolate SJ_92 scaffold_399 from base pairs 59364 to 62104 (sequence ID: JAOAMX010045185.1).

**SI Figure 5. Plasma paraments for dogfish exposed to acute low salinity and recovery in full-strength seawater.** *(****a****)* Plasma osmolality, *(****b****)* urea, and *(****c****)* glucose in dogfish (n = 5) before (0E) and after (48E) 48 h exposure to low salinity (21 ppt) followed by a 72 h recovery in full-strength seawater (32 ppt). Data are presented as means ± sem with individual values shown. Different letters indicate significant differences (*P* < 0.05) as determined using a linear mixed effects model where individual was assigned as a random variable.

(***b***)

(***a***)

**SI Figure 6. Plasma corticosterone concentrations during experiment 1 (low salinity) and experiment 2 (gavage).** (***a***) Dogfish (individuals denoted as distinct colours) were exposed to 21 ppt for 48 h (left of the dotted vertical line) and recovered for 72 h in 32 ppt seawater during which repeated blood samples were collected and plasma corticosterone measured. (***b***) Dogfish received a gavage of seawater (control; blue) or antibiotics cocktail (red) daily for 5 days followed by 72 h in individual recovery boxes. Plasma samples were collected upon entry (0 h) and exit (72 h) of the individual boxes. In both experiments, corticosterone was measured following HPLC separation using a commercial EIA. No significant effects of treatment or time were detected using a linear mixed effects model (*P* < 0.05).
